# Associations between biomarkers of multimorbidity burden and mortality risk among patients with acute dyspnea

**DOI:** 10.1007/s11739-021-02825-6

**Published:** 2021-08-20

**Authors:** Torgny Wessman, Rafid Tofik, Thoralph Ruge, Olle Melander

**Affiliations:** 1 Department of Emergency Medicine, Skåne University Hospital, Ruth Lundskogs gata 3, 20502 Malmö, Sweden; 2 Department of Internal Medicine, Skåne University Hospital, Malmö, Sweden; 3 Department of Clinical Sciences Malmö, Lund University, Malmö, Sweden

**Keywords:** Acute dyspnea, Emergency department, Biomarker, Comorbidity, Multimorbidity, METTS, Risk factors

## Abstract

The patients’ burden of comorbidities is a cornerstone in risk assessment, clinical management and follow-up. The aim of this study was to evaluate if biomarkers associated with comorbidity burden can predict outcome in acute dyspnea patients. We included 774 patients with dyspnea admitted to an emergency department and measured 80 cardiovascular protein biomarkers in serum collected at admission. The number of comorbidities for each patient were added, and a multimorbidity score was created. Eleven of the 80 biomarkers were independently associated with the multimorbidity score and their standardized and weighted values were summed into a biomarker score of multimorbidities. The biomarker score and the multimorbidity score, expressed per standard deviation increment, respectively, were related to all-cause mortality using Cox Proportional Hazards Model. During long-term follow-up (2.4 ± 1.5 years) 45% of the patients died and during short-term follow-up (90 days) 12% died. Through long-term follow-up, in fully adjusted models, the HR (95% CI) for mortality concerning the biomarker score was 1.59 (95% CI 1348–1871) and 1.18 (95% CI 1035–1346) for the multimorbidity score. For short-term follow-up, in the fully adjusted model, the biomarker score was strongly related to 90-day mortality (HR 1.98, 95% CI 1428–2743), whereas the multimorbidity score was not significant. Our main findings suggest that the biomarker score is superior to the multimorbidity score in predicting long and short-term mortality. Measurement of the biomarker score may serve as a biological fingerprint of the multimorbidity score at the emergency department and, therefore, be helpful for risk prediction, treatment decisions and need of follow-up both in hospital and after discharge from the emergency department.

**Supplementary Information:**

The online version contains supplementary material available at 10.1007/s11739-021-02825-6.

## Background

Shortness of breath, further on referred to as dyspnea, is one of the most common causes of visits to emergency departments [1]. Most patients initially present the symptom of dyspnea, rather than a predetermined diagnosis. The main underlying diagnosis is sometimes unclear during the first hours. Cardiovascular disease (CVD) and congestive heart failure (CHF) as well as chronic obstructive pulmonary disease (COPD), are some of the most common underlying causes of acute dyspnea [2–4]. All of these are diseases with sometimes a poor outcome and a high mortality. It is a challenge in the emergency department to quickly identify patients with more serious illness and with risk factors for poorer outcome and higher mortality. Evaluating the presence and numbers of comorbidities can provide important prognostic information. Today there is a great interest in research concerning on how novel biomarkers can provide new information about diseases, disease severity, risks and mortality. In this study the aims were to investigate if a biologic fingerprint in the form of a score of biomarkers associated with multimorbidity, can add independent information regarding long-term as well as short-term mortality risks in acute dyspnea patients on top of clinical information on multimorbidity and other known risk factors for mortality in this common emergency department patient group.

## Methods

### Study population and plan

For this study, 774 patients from the ADYS cohort, included from 6 March 2013 to 20 January 2016, and with complete analyzes of 80 biomarkers from the Olink CVD1 panel (Olink Proteomics AB, Sweden. See explanations in proteomics profiling section below and supplementary table 1), were included. Patients 18 years of age and older who presented to the emergency department during daytime, 6:45 a.m. to 4:30 p.m. working days, with acute dyspnea as their main complaint, were informed about the study, and asked for their written informed consent. This study complies with the Declaration of Helsinki. Critically ill patients who directly were transferred from the resuscitation room to an intensive care unit (ICU) were excluded, as were patients with lower degrees of consciousness, as these patients were not able to give consent nor to partake in an interview performed by a research nurse. A research nurse collected information from the patients’ medical hospital records, and patients were interviewed about their health, medication, symptoms, social situation, etc. according to a standardized and approved questionnaire (see supplemental Tables 2, 3). Vital parameters were registered as were medical triage priority level according to the validated Medical Emergency Triage and Treatment System (METTS) [5] and dyspnea severity using a similar scale as the NYHA classification [6]. Blood samples were drawn within an hour of presentation to the emergency department and then frozen within 2 h of collection and stored at − 80 °C until analysis.

In 2017, a data file containing data from these 774 patients was sent in a coded file to Statistiska Centralbyrån (SCB), the state agency for Statistics Sweden. At SCB the file was decoded, and SCB added information on an individual basis about annual incomes during the years 2012–2017. The file was then sent to the Swedish National Board of Health and Welfare (Socialstyrelsen, SoS), who used the code key from SCB to add information about cause of death until 31 December 2105 and date of death until 26 July 2017, i.e. end of the follow-up period. The file was returned to us from the Swedish National Board of Health and Welfare in such a way that all patients were anonymized. The cause of death register at SoS is updated more slowly than the all-cause death date register itself, which explains the differences in dates for death dates and causes of death. As our data file is anonymized, as required by Swedish law, it is not possible to afterwards supplement with later data from the Swedish Cause of Death Register.

### Proteomic profiling

The Olink Proseek^®^ Multiplex Cardiovascular I^96X96^ kit (http://www.olink.com/) is a proximity extension assay (PEA) that measures the relative abundance of cardiovascular proteins. Each analyte in the panel has according to Olink Proteomics AB been assessed in terms of sample material, specificity, precision, sensitivity, dynamic range, matrix effects and interference.

### Statistics

Our starting point was defining multimorbidity, as the presence of 22 predefined diseases and illnesses that were systematically registered by a nurse from the inclusion interview, complemented by searching medical records (Table 1). Each comorbidity was defined as evidence of prior or present existence of the comorbidity in question. Patients without affirmed evidence (negation or unknown) were defined as not having the specific comorbidity. By adding the number of comorbidities for each patient, a multimorbidity score was created, which was then standardized so that the score number corresponds to one SD increment for each comorbidity-number. We then tested associations between the 80 biomarkers using linear regression with a stepwise selection method together with age and gender, with the multimorbidity score as a continuous outcome (expressed on a standardized scale). Eleven biomarkers had a significant association with the multimorbidity score independently of each other and of age and sex. The biomarkers, individually weighted for their beta-coefficient in relation to the multimorbidity score (log-transformed and standardized), were then summed up to create the biomarker score (subsequently also log-transformed and put on a standardized scale). The biomarker score was expressed as number of standard deviations from the mean and used for further analyses in relation to mortality.

**Table 1:** Patient characteristics (*N* = 774)

Variable	Result
Age, years, mean (± SD)	70.6 (± 17.8)
Age > 65 years, *N* (%)	544 (70.3)
Sex (Male), *N* (%)	349 (45.1)
Affirmed comorbidities (previous/ongoing), *N* (%)	
Hypertension	343 (44.3)
Congestive heart failure	264 (34.1)
Coronary artery disease	253 (32.7)
Atrial fibrillation	228 (29.5)
Chronic obstructive pulmonary disease	225 (29.1)
Infection	221 (28.6)
Obesity	166 (21.4)
Anaemia	147 (19.0)
Diabetes	143 (18.5)
Cancer	136 (17.6)
Pulmonary embolism	92(11.9)
Asthma	83 (10.7)
Stroke	82 (10.6)
Renal disease	75 (9.7)
Restrictive pulmonary disease	39 (5.0)
Rheumatic disease	38 (4.9)
Depression	36 (4.7)
Anxiety	30 (3.9)
Hip fracture	27 (3.5)
Dementia	23 (3.0)
Other pulmonary disease	13 (1.7)
Neuromuscular disease	4 (0.5)
Smoking	
Ongoing or previous smoker, *N* (%)	536 (69.3)
Arrival mode	
Ambulance, *N* (%)	436 (56.3)
Alarm	77 (9.9)
Inhabitant of Malmö, *N* (%)	697 (90.1)

Data are presented as median (interquartile range) or mean (± SD), depending on the presence or absence of normal distribution of data. Group-wise differences of continuous variables were compared using ANOVA or Kruskal–Wallis test when appropriate. Categorical variables were compared between groups using chi-squared test.

We used Cox proportional hazards model to relate the biomarker score to mortality during the entire follow-up (long-term) and a fixed 90-day follow-up period (short-term). We used three models of adjustment in the analyses relating the biomarker score with mortality: (model 1) age and sex, (model 2) age, gender and the multimorbidity score and (3) age, gender, the multimorbidity score, METTS triage priority, dyspnea level, annual income and smoking. A two-tailed significance level of (*p* < 0.05) was considered statistically significant. All calculations were done with IBM SPSS Statistics version 25.0.

## Results

### Baseline characteristics

In our cohort the mean age was 70 years, 70% of the patients were > 65 years and 45% were male, 69% were previous or ongoing smokers and 56% arrived at the emergency department with an ambulance (Table 1). For the actual distribution of the 22 different comorbidities see Table 1. The mean number of comorbidities were 3.4 (SD ± 2.4) with the three most common being hypertension (44.3%), CHF (34.1%) and CAD (32.7%). In Table 2, we show clinical baseline characteristics of our cohort stratified into four groups based on increasing number of comorbidities (0–1 comorbidities, 2–3 comorbidities, 4–5 comorbidities and 6 or more comorbidities (Table 2). There were significant linear associations between increasing number of comorbidities and METTS triage priority, dyspnea level and yearly earned income (Table 2).

**Table 2:** Baseline characteristics in comorbidity score groups (*N* = 774)

	0–1 comorbidity, *N* = 183	2–3 comorbidities, *N* = 233	4–5 comorbidities, *N* = 211	6 or more comorbidities, *N* = 147
Age (years) mean (± SD)	52.8 (± 19.5)	71.3 (± 15.3)	79.0 (± 10.8)	78.9 (± 9.2)
Male gender (*N* (%))	61 (33.3%)	114 (48.9%)	97 (46%)	77 (52.4%)
Earned income (SEK) the year prior to the inclusion, median (interquartile range)*	165,404 (122,828–267,721	153,249 (132,621–192,479)	147,672 (125,213–172,532)	142,910 (126,568–161,757)
Smoking ongoing and previous (*N* (%))*	119 (65%)	161 (69.1%)	152 (72%)	104 (70.7%)
METTS green, priority 4 (*N* (%))*	27 (14.8%)	13 (5.6%)	5 (2.4%)	5 (3.4%)
METTS yellow, priority 3 (*N* (%))	97 (53.0%)	119 (51.1%)	85 (40.3%)	59 (40.1%)
METTS orange, priority 2 (*N* (%))	48 (26.2%)	71 (30.5%)	87 (41.2%)	58 (39.0%)
METTS red, priority 1 (*N* (%))	10 (5.5%)	29 (12.4%)	33 (15.6%)	25 (17.0%)
Dyspnea level 1, no symptoms (*N* (%))*	86 (47.0%)	65 (27.9%)	32 (15.2%)	14 (9.5%)
Dyspnea level 2, mild symptoms (*N* (%))	57 (31.1%)	95 (40.8%)	85 (40.3%)	47 (32.0%)
Dyspnea level 3, marked limitation (*N* (%))	14 (7.7%)	31 (13.3%)	36 (17.1%)	34 (23.1%)
Dyspnea level 4, severe limitations (*N* (%))	21 (11.5%)	42 (18.0%)	57 (27.0%)	51 (34.7%)

*Missing values on 7 dyspnea level, 3 METTS priority, 2 ever smoke and 4 earned income

### Biomarkers in relation to multimorbidity and discharge diagnoses

The point estimate of the beta-coefficient of the biomarkers (expressed as per SD increment of log-transformed value of the biomarker in question) in relation to the multimorbidity score (expressed as per SD increment of the multimorbidity score) ranged from positive values between 0.206 and 0.494 and negative values between − 0.285 and (− 0.207) (Table 3). Three of the 11 independently significant biomarkers had negative relationships with the multimorbidity score [Platelet endothelial cell adhesion molecule (PECAM1), Interleukin 27A (IL27A), Galanin peptides (GAL)] and the three biomarkers with highest beta-coefficient per SD increment (regardless of directionality) were N-terminal prohormone of brain natriuretic peptide (NTproBNP), Fibroblast growth factor (FGF23) and Fatty Acid Binding Protein 4 (FAB4).

**Table 3:** Biomarkers significantly related to comorbidity score, age and sex adjusted

	Unstandardized coefficients		Standardized coefficients	*p* value
	*B*	Std. error		
NTproBNP, N-terminal prohormone of brain natriuretic peptide	0.494	0.091	0.209	< 0.0001
FGF23, Fibroblast growth factor	0.438	0.088	0.181	< 0.0001
FABP4, Fatty Acid Binding Protein 4	0.333	0.101	0.141	0.001
CCL20, C–C motif chemokine 20	0.286	0.076	0.120	0.0002
SCF, Stem cell factor	0.271	0.077	0.115	0.0004
REN, Renin	0.266	0.071	0.114	0.0002
LEP, Leptin	0.254	0.077	0.108	0.001
MMP12, Matrix Metallo-proteinase	0.206	0.078	0.086	0.008
IL27A, Interleukin 27A	− 0.207	0.076	− 0.085	0.006
PECAM1, Platelet endothelial cell adhesion molecule	− 0.212	0.069	− 0.090	0.002
GAL, Galanin peptides	− 0.285	0.076	− 0.119	0.0002

A linear stepwise regression of all the discharge diagnoses as well as age and gender as independent variables and the biomarker score as dependent, showed that the discharge diagnose heart failure [0.51 ± 0.075 standard error (SE) per increment of the biomarker score] and the discharge diagnose thromboembolic disease (0.37 ± 0.17 SE per increment of the biomarker score) were significantly associated with the biomarker score.

### Biomarker score of comorbidities and actual multimorbidity in relation to mortality

As for the multimorbidity score (Table 2), the biomarker score (Table 4) showed significant linear associations with several of the baseline characteristics.

**Table 4:** Baseline characteristics in biomarker score quartiles (*N* = 774)

	Biomarker score Q1 (Low), *N* = 193	Biomarker score Q2, *N* = 194	Biomarker score Q3, *N* = 194	Biomarker score Q4 (High), *N* = 193	*p* value (linear-by-linear)
Age (years) mean (± SD)	507 (± 17.3)	73.2 (± 12.7)	78.0 (± 12.4)	80.4 (± 10.1)	< 0.0001
Male gender (*N* (%))	84 (43.5)	91 (46.9)	98 (50.5)	76 (39.4)	n.s
Earned income (SEK) the year prior to the inclusion. mean (± SD)**	198 866 (± 125 021)	180 974 (± 111 648)	171 545 (± 97 500)	156 587 (± 62 439)	< 0.0001
Smoking ongoing and previous (*N* (%))**	126 (65.3)	142 (73.6)	136 (70.1)	132 (68.0)	n.s
METTS green, priority 4 (*N* (%))**	24 (12.4)	14 (7.2)	8 (4.1)	4 (2.1)	< 0.0001
METTS yellow, priority 3 (*N* (%))	115 (59.6)	87 (44.8)	84 (43.3)	74 (38.3)	
METTS orange, priority 2 (*N* (%))	42 (21.8)	71 (36.6)	77 (39.7)	74 (38.3)	
METTS red, priority 1 (*N* (%))	11 (5.7)	21 (10.8)	24 (12.4)	41 (21.2)	
Dyspne level 1, no symptoms (*N* (%))**	96 (49.7)	54 (27.8)	31 (16.0)	16 (8.3)	< 0.0001
Dyspne level 2, mild symptoms (*N* (%))	59 (30.6)	70 (36.1)	77 (39.7)	78 (40.4)	
Dyspne level 3, marked limitation (*N* (%))	15 (7.8)	33 (17.0)	40 (20.6)	27 (14.0)	
Dyspne level 4, severe limitations (*N* (%))	18 (9.3)	37 (19.1)	44 (22.7)	72 (37.3)	

*BMS = biomarker-multimorbidity score

**Missing values on 7 dyspnea level, 3 METTS priority, 2 ever smoke and 4 earned incomes

The biomarker score, based on the 11 biomarkers independently associated with the multimorbidity score, was subsequently related to risk of mortality during long- and short-term follow-up.

In our primary outcome, at long-term follow-up (2.4 ± 1.5 years) 348 patients died (45%). In model 1 (adjusted for age and gender), there was a significant increase of death per each SD increment of the biomarker score with 60% and for the multimorbidity score 43% (Table 5). When the biomarker score and the multimorbidity score were entered simultaneously together with age and sex (model 2), the mortality risk per SD increment of the biomarker score was 59% and for the multimorbidity score it was 18% (Table 5).

**Table 5:** Biomarker score and multimorbidity score in single*, partly** and fully*** adjusted models for long-term and 90-days follow-up, *N* = 774

	HR	95% CI	*p* value
Long term follow-up			
Single model			
Multimorbidity score	1.427	1.275–1.597	< 0.0001
Biomarker score	1.763	1.532–2.028	< 0.0001
Single model partly adjusted			
Multimorbidity score	1.180	1.035–1.346	0.013
Biomarker score	1.588	1.348–1.871	< 0.0001
Fully adjusted model			
Multimorbidity score	1.105	0.965–1.266	n.s
Biomarker score	1.533	1.299–1.810	< 0.0001
90-days follow-up			
Single model			
Multimorbidity score	1.179	0.945–1.471	n.s
Biomarker score	1.847	1.407–2.425	< 0.0001
Single model partly adjusted			
Multimorbidity score	0v883	0.681–1.146	n.s
Biomarker score	1.998	1.456–2.742	< 0.0001
Fully adjusted model			
Multimorbidity score	0.843	0.641–1.109	n.s
Biomarker score	1.979	1.428–2.743	< 0.0001

*Adjusted for age and gender

**Adjusted for age, gender, biomarker score and multimorbidity score independently

***Adjusted for age, gender, biomarker score, multimorbidity score, annual income, ever smoker, METTS priority and dyspnea level

In a fully adjusted multivariate model (Table 5), adjusting for age and gender, the biomarker score, the multimorbidity score and all the significant variables from our previous study on the ADYS cohort independently (METTS triage priority, dyspnea level, annual income and smoking) [7] there was a significant increase of mortality risk for the biomarker score with 55% per SD increment, whereas the multimorbidity score was not significantly related to mortality*.* After adjustment for NTproBNP or C-reactive protein, or removal of NTproBNP from the biomarker score the biomarker score still remained significantly related to mortality (data not shown).

As a secondary outcome, we investigated the association between the biomarker score and death during short-term follow-up (90-days). At short-term follow-up 94 of patients had died (12%). The biomarker score in the fully adjusted model significantly predicted a 98% increase of short-term mortality per each SD increment, whereas the multimorbidity score was not significantly related to short-term mortality (Table 5).

In analyses of quartiles in a fully adjusted model, there was a significant linear trend between the biomarker score quartiles (uncategorized) and mortality at long-term (*p* ≤ 0.0001), and at short-term follow-up (*p* = 0.001). With the biomarker score categorized, patients in the top vs bottom quartile had a 2.3-fold increased risk of death for long-term and a 4.2-fold increase in short-term mortality (Table 6).

**Table 6:** Biomarker score quartiles in fully adjusted* models for full and 90-days follow-up time, *N* = 774

	Number of deaths	HR	95% CI	*p* value
Long term follow-up time				
Biomarker score Q1 (low), *n* = 193	20	Ref		
Biomarker score Q2, *n* = 194	76	1.535	0.891–2.645	n.s
Biomarker score Q3, *n* = 194	104	1.854	1.069–3.214	0.028
Biomarker score Q4 (high), *n* = 193	148	2.942	1.667–5.193	0.0002
90-days follow-up time				
Biomarker score Q1 (low), *n* = 193	4	Ref		
Biomarker score Q2, *n* = 194	19	2.009	0.550–7.331	n.s
Biomarker score Q3, *n* = 194	21	2.210	0.599–8.159	n.s
Biomarker score Q4 (high), *n* = 193	50	4.700	1.265–17.466	0.021

*Adjusted for age, gender, biomarker score, multimorbidity score, annual income, ever smoker, METTS priority and dyspnea level

## Discussion

In this study we have demonstrated that the use of a score of 11 different biomarkers associated with multimorbidity strongly enhances the mortality risk stratification in patients seeking care because of acute dyspnea, besides information on aggregated number of comorbidities, smoking habits annual income, medical triage priority according to METTS and dyspnea severity measured according to NYHA classification.

Medical history regarding the presence of comorbidities is a cornerstone not only for risk stratification and management in the acute situation at the emergency department but also for determination of level of care and follow-up after dischargeIn the emergency department, the primary focus is to take care of the emergent situation, with management and treatments of immediate risks and symptom relief. Whereas the presence of individual comorbidities may guide the emergency department physician to the likely cause of an acute event, risk stratification for fatal outcome related to the total burden of comorbidities in the form of comorbidity indices might be more informative. In population epidemiology, the most common comorbidity scores are Charlson Comorbidity Index (CCI) [8, 9] and Elixhauser comorbidity measure (ECM) [10] for predicting mortality and health status [11–13]. However, these are to our knowledge not validated for the use in an emergency department.

With our approach, we had to exclude unconscious and seriously ill patients for ethical reasons because they could not get information and give their consent to the study. In any case, we believe that the most severely ill and unconscious patients would usually get high medical attention and high level of care regardless of a biomarker test. It is our opinion the biomarker score could aid in the challenge to find the patients with severe disease among the remaining patients as well as to possibly rule out severe disease to safely send home some patients.

The use of biomarkers in the acute patient care setting is an interesting and important emerging issue. As a complement to comorbidity indices and medical triage, the use of biomarkers for evaluation and risk stratification can be cost effective, safe and a fast test in the emergency department setting.

In this study, instead of examining the association of one biomarker at a time regarding outcomes, we hypothesized that the aggregated information of a broad set of circulating protein biomarkers in blood, previously implicated in cardiovascular disease [14–17], would serve as a biological fingerprint of multimorbidity in acute dyspnea patients. It is important to underline that the strategy we undertook was to first identify a biological fingerprint (the biomarker score) of multimorbidity burden and then to test if such a comorbidity related biological fingerprint was a better predictor of mortality than the actual comorbidities themselves. This strategy reduced the risk of data-driven results, as would be expected to have been a problem if we had directly related all 80 biomarkers with mortality. We did see strong crude correlations between the biomarker score and several baseline characteristics, which previously have been linked to mortality risk in acute dyspnea, however, when these were adjusted for, robust relationships between the biomarker score and mortality remained. Presumably, the superiority of biomarker scores versus comorbidity score in predicting death, partly could be explained by the dynamic nature of blood biomarkers. Apart from reflecting biological burden due to chronic disease, the biomarker score is probably also affected by acute disease states. Partly the biomarkers in the biomarker score are related to a broad range of physiological and pathophysiological processes such as hemodynamics, inflammation, metabolism and tissue repair. The various pathways involved and expressed in the biomarker score, probably better reflect the complex and multifactorial nature of acute dyspnea and the comorbidities present in such patients. Of note, even if NTproBNP, which is commonly taken as routine in acute dyspnea, was one of the eleven biomarkers included on the biomarker score, a sensitivity analysis showed that the biomarker score remained strongly related to mortality even when NTproBNP was removed or removed and adjusted for.

C-reactive protein (C-RP) is a common blood test which is more or less a standard test at an emergency department as a marker of severity. There are studies showing an association between raised levels of C-RP and cardiovascular disease [18]. However, in this study, the biomarker score remained highly significant also after additional adjustment for C-RP).

Most emergency department studies restrict endpoint follow-up to 30 or 90 days as any events occurring later are less likely to be related to the acute event that brought the patient to the emergency department. Although this is true, it is also important to consider the risk of mortality in the long run, to be able to plan the need of follow-up when the patient is discharged from the emergency department or the hospital ward. In our study we followed our patients for an average of 2.4 years, assuming that the risk of death during this long-term follow-up time would be of relevance for the planning of health care follow-up. The hazard ratio for death conferred by the biomarker score was greater during short-term than long-term follow-up, again indicating influence of the acute conditions on the biomarker score. The hazard ratio for death attributable to the multimorbidity score, on the other hand, was greater during long-term than short-term follow-up, even if it was inferior to the biomarker score in both. The presence of the multimorbidity score is probably more important when diseases are allowed to act for a longer period of time, while blood proteins and biomarkers also reflects rapid dynamic changes.

From our and other studies, it appears that the estimation of risk with biomarkers reflecting comorbidity (the biomarker score) would add substantially more clinically relevant information concerning the primary risk assessment over short time for the emergency department physician compared to the multimorbidity score.

In the future, it could be possible to produce a combined point-of-care test with a selection of biomarkers, which identifies cut-off values of clinical significance, perhaps graded in quartiles. Such a point-of-care test, which could provide results for example low risk, intermediate risk, high risk and extremely high risk, could do so to a lower cost than generating 11 individual biomarker concentrations. Regarding the cost, this would be dependent on commercialization and the market request of such a test. More research and development of a point-of-care test is needed before our results could be translated into clinical practice.

### Strengths and limitations

Patients with high acuity or deranged consciousness could not give informed consent and went directly to the ICU and were, therefore, not included in this study.

The presence of comorbidities (past or present) where both asked for and the research nurses checked the medical records at our hospital. However, we did not check up the national patient visit register at SoS, thus possibly missing information of comorbidities from medical records and visits in other regions in Sweden. In the ADYS cohort, we have registered 22 different comorbidities. There may be other comorbidities that we have not registered, which is a limitation. We do, however, believe that we have captured the majority of important comorbidities when it comes to acute dyspnea patients.

It is a limitation that we did not use validated comorbidity index like CCI or ECM, which are used internationally for risk stratification and as references when evaluating the use of biomarkers for risk stratification in an emergency department.

Even though our study included almost 800 patients with analysed biomarkers, an even larger study cohort would be desirable to be able to detect exposures with smaller effect sizes.

Even if the blood samples used for later measurement of the biomarker score were drawn at the immediate entry to the emergency department, it is a strength that the results were not known for the emergency department physician and thus did not affect clinical decisions taken.

### Clinical implication

Medical history taking regarding the patient’s comorbidities is one of the fundaments for risk stratification both in the short- and long-term. Since the biomarker score is superior to medical history in predicting death, we argue that a simple blood test could be a valuable, quick, safe and hopefully cheap future complement for the emergency department physician.

## Conclusion

The biomarker score seems to be an independent risk factor for both short- and long-term mortality, not only independent of actual comorbidities but also independent of information about other risk factors as annual income, medical triage, smoking and dyspnea severity.

Our findings encourage both studies evaluating the relationship between the biomarker score and clinically relevant outcomes and randomized controlled trials testing if knowledge of the patients’ biomarker score versus no knowledge of the biomarker score improves care at the emergency department and during follow-up to a degree that mortality, functional status and quality of life is positively affected.

## Supplementary Information

Below is the link to the electronic supplementary material.

Supplementary file1 (DOCX 29 KB)

## Data Availability

The datasets generated during and/or analysed during the current study are available from the corresponding author on reasonable request.

## Abbreviations

ADYS: Acute dyspnea study
AF: Atrial fibrillation
CAD: Coronary artery disease
CVD: Cardiovascular disease
CHF: Congestive heart failure
COPD: Chronic obstructive pulmonary disease
ED: Emergency department
HF: Heart failure
ICU: Intensive care unit
IQR: Interquartile range
METTS: Medical Emergency Triage and Treatment System
SCB: Statistiska Centralbyrån, the state agency for Statistics Sweden
SES: Socioeconomic status
SoS: Socialstyrelsen, the Swedish National Board of Health and Welfare
SUS: Skånes Universitets Sjukhus, University Hospital of Skane
