# Supplementary material for: Associations between biomarkers of multimorbidity burden and mortality risk among patients with acute dyspnea
Source: Intern Emerg Med. Author manuscript; Available in PMC 2022 Mar 31. (PMC8964555; doi:10.1007/s11739-021-02825-6)
Supplement: Supplementary Material [file EMS140812-supplement-Supplementary_Material.docx]

**Supplementary table 1: Biomarkers n=80**

| AGRP | Agouti-related protein |
| --- | --- |
| AM | Adrenomedullin |
| CA125 | Ovarian cancer-related tumor marker CA 125 |
| CASP8 | Caspase-8 |
| CCL20 | C-C motif chemokine 20 |
| CCL4 | C-C motif chemokine 4 |
| CD40 | Tumor necrosis factor receptor superfamily member 5 |
| CD40L | CD40 ligand |
| CHI3L1 | Chitinase-3-like protein 1 |
| CSF1 | Macrophage colony-stimulating factor 1 |
| CSTB | Cystatin-B |
| CTSD | Cathepsin D |
| CTSL1 | Cathepsin L1 |
| CX3CL1 | Fractalkine |
| CXCL1 | C-X-C motif chemokine 1 |
| CXCL16 | C-X-C motif chemokine 16 |
| CXCL6 | C-X-C motif chemokine 6 |
| Dkk1 | Dickkopf-related protein 1 |
| ECP | Eosinophil cationic protein |
| EGF | Epidermal growth factor |
| ESM1 | Endothelial cell-specific molecule 1 |
| FABP4 | Fatty acid-binding protein, adipocyte |
| FAS | Tumor necrosis factor receptor superfamily member 6 |
| FGF23 | Fibroblast growth factor 23 |
| FS | Follistatin |
| GAL | Galanin peptides |
| Gal3 | Galectin-3 |
| GDF15 | Growth/differentiation factor 15 |
| GH | Growth hormone |
| HBEGF | Heparin-binding EGF-like growth factor |
| HGF | Hepatocyte growth factor |
| hK11 | Kallikrein-11 |
| HSP27 | Heat shock 27 kDa protein |
| IL8 | Interleukin-8 |
| IL16 | Interleukin-16 |
| IL18 | Interleukin-18 |
| IL1ra | Interleukin-1 receptor antagonist protein |
| IL27A | Interleukin-27 subunit alpha |
| IL6 | Interleukin-6 |
| IL6RA | Interleukin-6 receptor subunit alpha |
| KLK6 | Kallikrein-6 |
| LEP | Leptin |
| LOX1 | Lectin-like oxidized LDL receptor 1 |
| MB | Myoglobin |
| MCP1 | Monocyte chemotactic protein 1 |
| MMP10 | Matrix metalloproteinase-10 |
| MMP12 | Matrix metalloproteinase-12 |
| MMP7 | Matrix metalloproteinase-7 |
| MPO | Myeloperoxidase |
| NEMO | NF-kappa-B essential modulator |
| NTproBNP | N-terminal pro-B-type natriuretic peptide |
| OPG | Osteoprotegerin |
| PAPPA | Pappalysin-1 |
| PAR1 | Proteinase-activated receptor 1 |
| PDGFsubunitB | Platelet-derived growth factor subunit B |
| PECAM1 | Platelet endothelial cell adhesion molecule |
| PIGF | Placenta growth factor |
| PRL | Prolactin |
| RAGE | Receptor for advanced glycosylation end products |
| REN | Renin |
| RETN | Resistin |
| SCF | Stem cell factor |
| SELE | E-selectin |
| SIRT2 | SIR2-like protein 2 |
| SPON1 | Spondin-1 |
| SRC | Proto-oncogene tyrosine-protein kinase Src |
| ST2 | ST2 protein |
| TF | Tissue factor |
| TIE2 | Angiopoietin-1 receptor |
| TIM | TIM-1, T-cell immunoglobulin and mucin domain |
| TM | Thrombomodulin |
| TNFR1 | Tumor necrosis factor receptor 1 |
| TNFR2 | Tumor necrosis factor receptor 2 |
| TNFSF14 | Tumor necrosis factor ligand superfamily member 14 |
| tPA | Tissue-type plasminogen activator |
| TRAIL | TNF-related apoptosis-inducing ligand |
| TRAILR2 | TNF-related apoptosis-inducing ligand receptor 2 |
| UPAR | Urokinase plasminogen activator surface receptor |
| VEGFA | Vascular endothelial growth factor A |
| VEGFD | Vascular endothelial growth factor D |

**Supplementary table 2: ADYS questionnaire-basis for patient interview**

|  | **Living in Malmö**: 1-Yes, 2-No, 9-Unknown |
| --- | --- |
|  | **If Malmö, which suburban district**: 1-Centrum, 2-Södra Innerstaden, 3-Västra Innerstaden, 4-Limhamn Bunkeflo, 5-Hyllie, 6-Fosie, 7-Oxie, 8-Rosengård, 9-Husie, 10-Kirseberg, 11-Unknown |
|  | **Preliminary symptom at arrival/cause of visit**: 1-Dyspnea, 2-CHF, 3-CAD, 4-COPD/Asthma, 5-Pneumonia, 6-Tromboembolic disease, 7- Anxiety, 8-Other diagnosis, 9-Unknown. |
|  | **Manner of visit**:1- Own initiative, 2-Referral from Primary health care, 3-Referral from other, 9-Unknown |
|  | **If referral from other, state whom**: |
|  | **Civil status**: 1- married/partner, 2- partner with separate accommodation, 3-single, 9-unknown |
|  | **Heredity**: 1-cardiovascular disease, 2-pulmonary disease, 3- rheumatic disease, 4-cancer, 5-other, 9-unknown |
|  | **State profession**: |
|  | **Home medical health care**: 1-yes, 2-no, 9-unknown |
|  | **Home care**: 1-yes, 2-no, 9-unknown |
|  | **Technical aids in the home**: 1-yes, 2-no, 9-unknown |
|  | **Nursing home**: 1-yes, 2-no, 9-unknown |
|  | **Close relative in Scania**: 1-yes, 2-no, 9-unknown |
|  | **Need for interpreter**: 1-yes, 2-no, 9-unknown |
|  | **Ethnical background**; born in 1-Sweden, 2-Europe both not Sweden, 3-Outside of Europe |
|  | **Smoker**: 1-never smoked, 2-previous smoker, terminated >1 month, 3-smoker (party smoker) > 1month, 9-unknown |
|  | **Passiv smoker**: 1-yes, 2-no, 3-prior > 1 month, 9-unknown |
|  | **Amount of alcohol per week**:1-none, 2-less than 4 glasses (20 cl) wine (or corresponding amount beer/liquor) per week, 3- more than 4 glasses (20 cl) wine (or corresponding amount beer/liquor) per week, 4-somatic signs of alcoholism, 9-unknown |
|  | **Drug abuse**: 1-yes, 2-no, 9-unknown |
|  | **Need for social care planning**: 1-yes, 2-no, 9-unknown |
|  | **Previous/ongoing coronary heart disease (CHD**): 1-yes, 2-no, 9-unknown |
|  | **Previous/ongoing congestive heart failure (CHF**): 1-yes, 2-no, 9-unknown |
|  | **Previous/ongoing atrial flutter/fibrillation**: 1-yes, 2-no, 9-unknown |
|  | **Previous/ongoing Chronic Obstructive Pulmonary Disease (COPD):** 1-yes, 2-no, 9-unknown |
|  | **Previous/ongoing Asthma**: 1-yes, 2-no, 9-unknown |
|  | **Previous/ongoing Restrictive pulmonary disease**: 1-yes, 2-no, 9-unknown |
|  | **Previous/ongoing Other pulmonary disease**: 1-yes, 2-no, 9-unknown |
|  | **Previous/ongoing Neuromuscular disease (post-polio syndrome included)**: 1-yes, 2-no, 9-unknown |
|  | **Previous/ongoing Rheumatic disease**: 1-yes, 2-no, 9-unknown |
|  | **Previous/ongoing Pulmonary embolus/thromboembolic disease:** 1-yes, 2-no, 9-unknown |
|  | **Previous/ongoing Pneumonia or other serious infections requiring hospitalization**: 1-yes, 2-no, 9-unknown |
|  | **Previous/ongoing Anaemia (women Hb<115g/L, men <130g/L**): 1-yes, 2-no, 9-unknown |
|  | **Previous/ongoing Cancer**: 1-yes, 2-no, 9-unknown |
|  | **Previous/ongoing Obesity (BMI>30 kg/m^2^):** 1-yes, 2-no, 9-unknown |
|  | **Previous/ongoing Diabetes mellitus**: 1-yes, 2-no, 9-unknown |
|  | **Previous/ongoing Hypertension**: 1-yes, 2-no, 9-unknown |
|  | **Previous/ongoing Stroke or Transitory Ischemic Attack**: 1-yes, 2-no, 9-unknown |
|  | **Previous/ongoing Dementia**: 1-yes, 2-no, 9-unknown |
|  | **Previous/ongoing Anxiety disorder**: 1-yes, 2-no, 9-unknown |
|  | **Previous/ongoing Depression**: 1-yes, 2-no, 9-unknown |
|  | **Previous/ongoing Parenchymatic Kidney Disease**: 1-yes, 2-no, 9-unknown |
|  | **Previous/ongoing Hip Fracture**: 1-yes, 2-no, 9-unknown |
|  | **State previous/ongoing other disease**: |
|  | **Medication with ACE inhibitor**: 1-yes, 2-no, 9-unknown |
|  | **Medication with anticoagulant**: 1-yes, 2-no, 9-unknown |
|  | **Medication with A2 inhibitor**: 1-yes, 2-no, 9-unknown |
|  | **Medication with antipsychotics**: 1-yes, 2-no, 9-unknown |
|  | **Medication with antidepressant**: 1-yes, 2-no, 9-unknown |
|  | **Medication with acetyl salicylic acid**: 1-yes, 2-no, 9-unknown |
|  | **Medication with other platelet inhibitor (i.e. Clopidogrel**): 1-yes, 2-no, 9-unknown |
|  | **Medication with betablocker**: 1-yes, 2-no, 9-unknown |
|  | **Medication with calcium inhibitor**: 1-yes, 2-no, 9-unknown |
|  | **Medication with insulin**: 1-yes, 2-no, 9-unknown |
|  | **Medication with oral diabetic medication**: 1-yes, 2-no, 9-unknown |
|  | **Medication with digitalis**: 1-yes, 2-no, 9-unknown |
|  | **Medication with diuretics**: 1-yes, 2-no, 9-unknown |
|  | **Medication with aldosterone inhibitor**: 1-yes, 2-no, 9-unknown |
|  | **Medication with pulmonary inhalators**: 1-yes, 2-no, 9-unknown |
|  | **Medication with corticosteroids**: 1-yes, 2-no, 9-unknown |
|  | **Medication with levothyroxine**: 1-yes, 2-no, 9-unknown |
|  | **Medication with long-acting nitroglycerin**: 1-yes, 2-no, 9-unknown |
|  | **Medication with anxiolytics**: 1-yes, 2-no, 9-unknown |
|  | **Medication with analgesics**: 1-yes, 2-no, 9-unknown |
|  | **State name of medications if other relevant medication**: |
|  | **State total number of drugs**: |
|  | **How/in what way is the medication administered**: 1- self, 2-relative,3-other, 4-unknown |
|  | **If other, state whom (e.g. district nurse)**: |

**Supplementary table 3: ADYS registered variables**

|  | **Visit date** (yyyy-mm-dd): |
| --- | --- |
|  | **Social security number**: |
|  | **Birth date** (yyyy-mm-dd): |
|  | **Gender**: 1-Male, 2-Female |
|  | **Arrival by ambulance**: 1-yes, 2-no, 9-unkown |
|  | **Ambulance alarm**: 1-yes, 2-no, 9-unkown |
|  | **METTS priority**: 1-red, 2-orange, 3-yellow, 4-green, 9-unknown |
|  | **If previous CHF, was echo ever performed**: 1-yes, 2-no, 9-unkown |
|  | **If previous echo, what was the result of EF**:1-normal (>50%), 2-slightly impaired (40-49%), 3-moderately impaired (30-39%), 4-severely impaired (<30%), 9-unknown |
|  | **If previous echo, sign of diastolic dysfunction**: 1-yes, 2-no, 9-unkown |
|  | **Height** (m): |
|  | **Weight** (kg): |
|  | **Body Mass Index** (kg/m^2^): |
|  | **Systolic blood pressure** (mmHg): |
|  | **Diastolic blood pressure** (mmHg): |
|  | **Heart rate** (bpm): |
|  | **Pulse oximetry** (%): |
|  | **If oxygen treatment, state how many liters**: |
|  | **Respiratory rate** (per minute): |
|  | **Body temperature** (C^o^): |
|  | **Pulmonary auscultation**:1-normal, 2-abnormal |
|  | **Level of shortness of breath/dyspnea**: 1-unaffected, 2-mild shortness of breath, 3-shortness of breath on exertion, 4-shortness of breath at rest |
|  | **ECG taken at the ED**: 1-yes, 2-no, 9-unkown |
|  | **ECG rhythm**: 1-sinus rhythm, 2-atrial fibrillation/flutter, 3-other, 9-unknown |
|  | **ECG QRS**: 1-normal, 2-pacemaker, 3-left bundle branch block, 4-Q-wave, 5- right bundle branch block, 6-other, 9-unknown |
|  | **ECG STT**: 1-normal, 2-ST-elevation, 3-ST-depression, 4-pathological T-wave, 5-other, 9-unknown |
|  | **Medication at ED** newly introduced and/or temporary treatment; **diuretics**: 1-yes, 2-no, 9-unkown |
|  | **Medication at ED** newly introduced and/or temporary treatment; **digitalis**:1-yes, 2-no, 9-unkown |
|  | **Medication at ED** newly introduced and/or temporary treatment; **nitrates**:1-yes, 2-no, 9-unkown |
|  | **Medication at ED** newly introduced and/or temporary treatment; **oxygen**:1-yes, 2-no, 9-unkown |
|  | **Medication at ED** newly introduced and/or temporary treatment; **continuous positive airway pressure**:1-yes, 2-no, 9-unkown |
|  | **Medication at ED** newly introduced and/or temporary treatment; **bronchodilator inhalations**:1-yes, 2-no, 9-unkown |
|  | **Medication at ED** newly introduced and/or temporary treatment; **corticosteroids**:1-yes, 2-no, 9-unkown |
|  | **Medication at ED** newly introduced and/or temporary treatment; **theophylline**:1-yes, 2-no, 9-unkown |
|  | **Medication at ED** newly introduced and/or temporary treatment;  **beta-2-stimulant injection**:1-yes, 2-no, 9-unkown |
|  | **Medication at ED** newly introduced and/or temporary treatment; **blood transfusion**: 1-yes, 2-no, 9-unkown |
|  | **Medication at ED** newly introduced and/or temporary treatment; **antibiotics**:1-yes, 2-no, 9-unkown |
|  | **Medication at ED** newly introduced and/or temporary treatment;  **if other** relevant medication state which: |
|  | **Examination at the ED** performed; **X-ray**:1-yes, 2-no, 9-unkown |
|  | Examination at the ED performed; **if chest x-ray state the result**:1-normal, 2-congestion, 3-pleural effusion, 4-parenchymal infiltration (incl. suspect TB & malignancy)5- pneumothorax, 6-other, 9-unknown |
|  | **Examination at the ED performed; echo**:1-yes, 2-no, 9-unkown |
|  | Examination at the ED performed; **if echo state the result of EF** :1-normal (>50%), 2-slightly impaired (40-49%), 3-moderately impaired (30-39%), 4-severely impaired (<30%), 9-unknown |
|  | Examination at the ED performed; **if echo, was the pressure in the pulmonary arteries** **elevated**:1-yes, 2-no, 9-unkown |
|  | Examination at the ED performed; **if echo, signs of diastolic dysfunction**:1-yes, 2-no, 9-unkown |
|  | **Examination at the ED performed; computer scan of thorax**:1-yes, 2-no, 9-unkown |
|  | Examination at the ED performed; **if computer scan of thorax, state the main result**: 1-normal, 2-congestion, 3-pleural effusion, 4-malignancy, 5-pneumonia, 6-pulmonary embolus, 7-pneumothorax, 8-other pulmonary disease, 9-unknown |
|  | **Examination at the ED performed; pulmonary scintigraphy**:1-yes, 2-no, 9-unkown |
|  | Examination at the ED performed**; if pulmonary scintigraphy, state the result**: 1- probable pulmonary embolism, 2-other diagnose (incl. pulmonary embolism cannot be ruled out), 9-unknown |
|  | **Examination at the ED performed; stress test of the heart:**1-yes, 2-no, 9-unkown |
|  | Examination at the ED performed; **if stress test of the heart, which**: 1-exertion test, 2- myocardial scintigraphy, 3-stress echocardiography |
|  | Examination at the ED performed; **if stress test of the heart, state the result**: 1-normal, 2-pathological, 3-not assessable, 9-unknown |
|  | **Examination at the ED performed; x-ray/angiography of coronaries**:1-yes, 2-no, 9-unkown |
|  | Examination at the ED performed; **if other relevant examinations** state what: |
|  | **CRP** (mg/L): |
|  | **Haemoglobin** count (g/L): |
|  | **White blood cells** (leukocytes) count (x10 9/L): |
|  | **Sodium** (mmol/L) |
|  | **Potassium** (mmol/L) |
|  | **Creatinine** (umol/L) |
|  | **Ionized calcium** (mmol/L) |
|  | **Troponin T** (ng/L) |
|  | **Glucose** (mmol/l) |
|  | **Venous pH** |
|  | **Venous pCO^2^** (kPa) |
|  | **Venous base excess** (mmol/l) |
|  | **Lactate** (mmol/L) |
|  | **Total carbon dioxide** (mmol/l) |
|  | **Is arterial blood gas taken**: 1-yes, 2-no, 9-unkown |
|  | **Biobank blood samples taken**: 1-yes, 2-no, 9-unkown |
|  | **Biobank DNA test taken**: 1-yes, 2-no, 9-unkown |
|  | **Admitted to hospital in ward**: 1-yes, 2-no, 9-unkown |
|  | If admitted to ward, **what type**: 1-internal medicine ward, 2-intensive care unit, 3-emergency ward, 4-cardiology ward, 5-pulmonary ward, 6-infection ward, 7other ward, 9-unknown |
|  | **If other ward, state which**: |
|  | **If discharged from hospital, to where:** 1-independent living, 2-temporary other accommodation, 3-permanent other accommodation, 4-another healthcare clinic, 5-other, 9-unknown |
|  | **Deceased during ED stay**: 1-yes, 2-no, 9-unkown |
|  | **First diagnose at discharge from ED**: 1-CHF, 2-acute coronary syndrome, 3-COPD/asthma, 4-pneumonia/other serious infection, 5-thromboembolic disease, 6-malignancy, 7-anxiety disorder, 8-other disease, 9-unknown |
|  | **If other first diagnose, state which**: |
|  | **Second diagnose at discharge from ED**: 1-CHF, 2-acute coronary syndrome, 3-COPD/asthma, 4-pneumonia/other serious infection, 5-thromboembolic disease, 6-malignancy, 7-anxiety disorder, 8-other disease, 9-unknown |
|  | **If other second diagnose, state which:** |
|  | **Third diagnose at discharge from ED**: 1-CHF, 2-acute coronary syndrome, 3-COPD/asthma, 4-pneumonia/other serious infection, 5-thromboembolic disease, 6-malignancy, 7-anxiety disorder, 8-other disease, 9-unknown |
|  | **If other third diagnose, state which**: |
|  | Fourth diagnose at discharge from ED: 1-CHF, 2-acute coronary syndrome, 3-COPD/asthma, 4-pneumonia/other serious infection, 5-thromboembolic disease, 6-malignancy, 7-anxiety disorder, 8-other disease, 9-unknown |
|  | **If other fourth diagnose, state which:** |
|  | **If other relevant diagnose at discharge from ED, state which?** |
|  | **Deceased during care in the hospital ward**: 1-yes, 2-no, 9-unkown |
|  | **First diagnose at discharge from hospital ward**: 1-CHF, 2-acute coronary syndrome, 3-COPD/asthma, 4-pneumonia/other serious infection, 5-thromboembolic disease, 6-malignancy, 7-anxiety disorder, 8-other disease, 9-unknown |
|  | **If other first diagnose, state which**: |
|  | **Second diagnose at discharge from hospital ward**: 1-CHF, 2-acute coronary syndrome, 3-COPD/asthma, 4-pneumonia/other serious infection, 5-thromboembolic disease, 6-malignancy, 7-anxiety disorder, 8-other disease, 9-unknown |
|  | **If other second diagnose, state which**: |
|  | **Third diagnose at discharge from hospital ward**: 1-CHF, 2-acute coronary syndrome, 3-COPD/asthma, 4-pneumonia/other serious infection, 5-thromboembolic disease, 6-malignancy, 7-anxiety disorder, 8-other disease, 9-unknown |
|  | **If other third diagnose, state which:** |
|  | **If other relevant diagnose at discharge from hospital ward, state which**: |

EF=ejection fraction; echo=echocardiogram; ECG=electrocardiogram; ED=emergency department; bpm=beats per minute; CHF=congestive heart failure;
